# Screening for latent tuberculosis infection in patients with chronic kidney disease: a review of evidence and current practice in the UK

**DOI:** 10.1093/ckj/sfaf197

**Published:** 2025-06-19

**Authors:** Joseph Sturman, Jyoti Baharani, Jed Ashman, Lorraine Harper, Adam F Cunningham, Martin Dedicoat, Matthew K O'Shea

**Affiliations:** Department of Immunology and Immunotherapy, School of Infection, Inflammation and Immunology, College of Medicine and Health, University of Birmingham, Birmingham, UK; Department of Renal Medicine, University Hospitals Birmingham NHS Foundation Trust, Birmingham, UK; Department of Renal Medicine, University Hospitals Birmingham NHS Foundation Trust, Birmingham, UK; Department of Renal Medicine, University Hospitals Birmingham NHS Foundation Trust, Birmingham, UK; Department of Renal Medicine, University Hospitals Birmingham NHS Foundation Trust, Birmingham, UK; Department of Applied Health Sciences, School of Health Sciences, University of Birmingham, Birmingham, UK; Department of Immunology and Immunotherapy, School of Infection, Inflammation and Immunology, College of Medicine and Health, University of Birmingham, Birmingham, UK; Department of Infectious Diseases, University Hospitals Birmingham NHS Foundation Trust, Birmingham, UK; Department of Immunology and Immunotherapy, School of Infection, Inflammation and Immunology, College of Medicine and Health, University of Birmingham, Birmingham, UK; Department of Infectious Diseases, University Hospitals Birmingham NHS Foundation Trust, Birmingham, UK

**Keywords:** chronic kidney disease, screening, tuberculosis

## Abstract

Tuberculosis (TB) and chronic kidney disease (CKD) represent an extant local and global syndemic, with TB incidence rates in the UK end-stage renal failure population far surpassing those of the general population in endemic countries. Patients with CKD generally have latent TB reactivation, rather than *de novo* infection, which presents with atypical, non-pulmonary presentations leading to late diagnosis, poorer outcomes and a high risk of widespread transmission through haemodialysis units. There is therefore a need to consider latent TB infection screening in the CKD population. However, there is widespread variation in local screening practices in the UK due to the challenge of diagnosing latent TB in CKD, and the absence of robust evidence-based guidelines. There is also concern that although a screening programme may have significant public health benefit, it may cause harm to the individual patient through adverse effects of treatment. In this review, we present the current evidence for latent TB infection screening in CKD, including the evidence of benefit and harm to the individual and the public. We also review current practices in the UK and present survey data from renal units in England demonstrating the diversity of policies currently in place. We advocate screening for latent TB in all CKD patients commencing dialysis and we highlight the pressing research questions that need to be urgently answered to help move towards a cohesive national policy to help drive evidence-based consistent care.

## INTRODUCTION

Tuberculosis (TB) and chronic kidney disease (CKD) represent an extant local and global syndemic of increasing concern, affecting the poorest and most vulnerable members of society [1–5]. Multiple previous studies have shown that the presence of CKD increases the risk of active TB disease, even when adjusting for age, ethnicity, country of birth and diabetes [2, 6–8]. Meta-analyses estimate the global incidence of TB in the dialysis population to be 5611 per 100 000 persons/year [1, 4], approximately 20 times higher than the incidence of TB in the general population of the World Health Organization's (WHO) South-East Asia region [3] (Table 1).

**Table 1:** Estimated incidence of active TB disease in different populations [1–4].

Population	TB incidence (per 100 000 persons/year)
WHO Europe region	25
WHO Africa region	208
WHO South-East Asia region	234
Global pre-dialysis CKD population	912
Global haemodialysis population	5611
Birmingham, UK haemodialysis population	256
Leicester, UK haemodialysis population	600

The effect of deteriorating renal function is stark.

The worldwide incidence of CKD also continues to rise and is predicted to become the fifth leading global cause of death by 2040, disproportionately affecting those living in lower-middle income countries, many of which have a high TB incidence [3, 9–11]. Nationally, the UK incidence of CKD is rising [12], and TB incidence has risen by 11% since 2022—the largest annual increase in incidence since current records began [13]. This means it is likely that there will be an increasing population that is at risk of simultaneous active TB disease and CKD. Consequently, it is of significant national and global importance to understand the best evidence-based practice for when to test, and how to treat, CKD patients with latent TB infection (LTBI) who often have multiple comorbidities. Patients with end-stage renal disease (ESRD) receiving in-centre haemodialysis are at particular risk of communicable diseases such as TB owing to the unavoidable necessity of attending a dialysis unit three times a week, where an outbreak would be hugely detrimental from both a healthcare and economic perspective.

This review aims to summarize the current challenges in diagnosing latent *Mycobacterium tuberculosis* (*Mtb*) infection in CKD/ESRD, and the evidence-base for deciding when to test and how to treat infection in this population. We also explore novel diagnostic approaches and future directions for research.

## THE CLINICAL COURSE OF TB DISEASE IN CKD

It is estimated that approximately 80% of active TB disease in the CKD cohort is attributed to reactivation of LTBI [5, 7, 14]. Immune dysregulation and impaired infection control is a hallmark of CKD more widely and specifically leads to an inability to control historic *Mtb* infections, leading to reactivation and symptomatic disease [15]. Importantly, there is a stepwise progression in the risk of active TB disease as renal function deteriorates [2, 8]. There is a particularly sharp increase in progression when CKD patients reach end ESRD and require dialysis. This is demonstrated by the dramatic 5-fold increase in the pooled global incidence of active TB disease in the haemodialysis population compared with the pre-dialysis CKD population (Table 1) [1]. Indeed, the global pooled prevalence of active TB disease in a systematic review and meta-analysis was higher in the haemodialysis group (34.8% of total haemodialysis population) than in the pre-dialysis CKD group (17.8%) or renal transplant population (16%) [16].

In addition to the increased risk of LTBI reactivation, clinical outcomes for treatment of active TB disease in CKD are worse. In a Chinese population of 117 CKD/haemodialysis patients with active TB, the hazard ratio for death relative to those with normal renal function was 4.7 (*P *= .018) if the patient had CKD Stage 4–5, and 6.1 (*P *= .005) if the patient was on haemodialysis [17]. One reason why outcomes may be poorer is because active TB disease commonly presents in an atypical fashion in advanced CKD. Extra-pulmonary TB represents 60%–80% of symptomatic presentations in CKD [1, 5, 17, 18]. As a result, the most common symptoms include fatigue, fevers, weight loss and anorexia, which are all symptoms that can be mistakenly attributed to CKD itself.

Consequently, not only does the risk of the development of active TB disease increase with CKD progression, but outcomes are also poorer [17, 18]. Furthermore, reactivation of LTBI and development of active pulmonary disease in patients that share dialysis facilities is a huge public health concern and previous documented outbreaks have required intensive contact tracing and treatment [19]. This would suggest there is a significant evidence base for testing and treating LTBI in CKD before the patient is established on haemodialysis. However, detecting and treating LTBI is extremely challenging in this patient population.

Some 2.8% of all new cases of TB in 2023 were in patients with CKD, representing 127 cases. It is difficult to calculate incidence as the UK Health Security Agency does not give details on the CKD stage of these cases. A data linkage study using data from the UK Renal Registry and the national Tuberculosis report would help to calculate accurate incidence rates. For comparison, patients are already screened based on country of birth, and the number of people from Bangladesh who had TB was similar at 144. If we assume the majority of the cases of TB in the CKD population were in the prevalent kidney replacement population, then the rate of TB in the dialysis population is 211/100 000 people compared with 22/100 000 in the total Bangladesh-born UK population [20, 21]. Given such a high number of cases in the CKD population, we advocate that, in the same way that country of origin is an established risk factor for the development of active TB disease, CKD patients should be identified as a high-risk group in which LTBI screening is appropriate [2, 4, 22, 23].

## CHALLENGES OF LTBI DIAGNOSIS IN CKD/ESRD

LTBI can be difficult to diagnose due to limitations of current diagnostic tests [24]. Current guidelines rely on the use of either the tuberculin skin test (TST) or interferon-gamma release assays (IGRA), which can also be positive in other mycobacterial infections such as *Mycobacterium bovis*. Further assessment then involves a detailed history and risk assessment plus a chest X-ray [5, 25]. This strategy has not changed in almost 14 years.

The issue with both TST and IGRA is that they measure the host immune response to purified antigens. In the context of CKD, which presents with a well-described immunosuppressive phenotype [15], the expected host immune responses may be absent, leading to false-negative or indeterminate results. Indeed, the sensitivity of TST in CKD has been shown to be extremely poor, with estimates between 20% and 30% [24, 26–28]. The specificity is also relatively unsatisfactory estimated to be between 9% and 63% [27, 28]. For reference, the TST in young, healthy, immunocompetent adults has a sensitivity of 94% and specificity of 88% [29]. Interestingly, IGRA tests perform slightly better in CKD: a previous systematic review and meta-analysis concluded that the QuantiFERON-TB Gold test had a pooled sensitivity of 53% (46%–59%) and specificity of 69% (65%–72%), while the T-SPOT.TB test had a pooled sensitivity of 50% (42%–49%) and specificity of 67% (61%–73%) [28, 30]. This is hypothesized to be because the IGRA test is an *in vitro* assay and therefore immune cells are removed from the uraemic extracellular environment of the patient, and so can function more appropriately [28]. Nevertheless, the sensitivity and specificity still leave much to be desired and highlights the difficulty in identifying cases in CKD/ESRD.

England's current LTBI screening programme advocates a single test without repeat testing [31]. Additionally, the WHO advises all dialysis patients undergo LTBI screening and they do not make a recommendation to repeat IGRA/TST testing [32]. In British Columbia, Canada, where there is a comprehensive LTBI screening programme in the dialysis population, repeat IGRA testing is also not recommended [33]. The European Centre for Disease Prevention and Control (ECDC) also does not recommend repeat IGRA testing in at risk groups including dialysis patients [34]. There have been attempts by some researchers to correct IGRA test results for serum creatinine levels which increased the number of positive results by 23% [35], but to date there are no studies evaluating repeat IGRA testing. It has been suggested that IGRA test results remain variable in CKD due to immune dysregulation [36]. This dysfunction is persistent and there is therefore no biological basis for repeat IGRA testing, unless the immune dysfunction could be reversed, which is an area of active research [32].

### When and who to test for LTBI

In the context of increased risk of TB and the challenges of diagnosing LTBI among CKD patients, consideration must be given on testing strategies.

Firstly, it is only cost-effective and ethical to test patients eligible for subsequent treatment. CKD-associated LTBI is normally treated in the UK with either a course of isoniazid and pyridoxine for 6 months, or with rifampicin, isoniazid and pyridoxine for 3 months, which can have adverse effects including peripheral neuropathy and hepatotoxicity [24, 25]. The rates of these adverse effects generally increase with age, with caution taken when considering treating older patients, although this convention is being challenged with more consideration given to active treatment for TB in the elderly [37]. The median age of the UK population on in-centre haemodialysis is 65.8 years, representing a relatively older cohort who would be more at risk of the adverse effects of treatment [38]. The question is whether the benefits of treating LTBI outweigh the risks of not doing so [32].

### Cost-effectiveness

A Markov statistical model has previously been developed to estimate the number of patients needed to screen (NNS) to prevent one case of active TB and the number of patients needed to treat (NNT) to prevent one death depending on various baseline comorbidities [24, 39]. For example, in the general population 2000–7000 people would have to be screened to prevent a single case, and up to 600 000 people would have to be treated to prevent one death. The benefit of screening and treatment seems greatest in patients with ESRD undergoing renal transplantation (NNS 70–250; NNT 300–1200). The NNS with ESRD on dialysis has been estimated as 140–5000, and the NNT 600–23 000. The very wide confidence interval for NNT means the benefit of treatment in the haemodialysis group is uncertain.

To compare with another well-known screening programme, the number of women needed to screen with mammography to prevent one case of breast cancer is 570, and to prevent one death is 2970 [40]. These figures are within the same range as those identified for LTBI screening, but it is important to remember that, as a communicable disease, screening for LTBI has wide-ranging public health implications and costs not captured by NNS/NNT, and therefore comparing with cancer screening programmes has limited merit. When considering the cost of contact tracing an entire dialysis unit in the event of a single case of active TB and implementing the appropriate isolation measures, there are significant health economic benefits to LTBI screening which are not easily captured by NNS/NNT figures.

To date, there is no cost-effectiveness analysis for LTBI screening specifically in the UK dialysis population. Nevertheless, much information can be extrapolated from such analysis that led to the introduction of the national LTBI testing and treatment programme for migrants [41]. The UK LTBI screening programme tests all new entrant migrants to England who arrive from a high TB incidence country (>150 cases per 100 000), have been in the UK <5 years and are aged 18–35 years [42]. In 2023, there was a total of 302 782 eligible individuals, but only 34 680 were tested (11.5%) and 5247 were positive (15.1%). Only 750 IGRA-positive individuals completed prophylactic treatment, representing 0.25% of the eligible population. One of the challenges for screening this population is they are young, highly mobile and asymptomatic, meaning that LTBI treatment compliance is understandably low. In contrast, 8254 people commenced kidney replacement therapy in 2022, the estimated incidence of LTBI in the dialysis population is 25–34.4% [22, 23], and TB incidence in the UK dialysis population is estimated to be between 256 and 600 per 100 000 person years [2, 4]. This is a significantly smaller, static population (due to the constraints of dialysis), who are therefore much easier to screen and monitor for treatment compliance than the migrant population. The dialysis population has a TB incidence significantly greater than the threshold incidence for country of origin, supporting cost-effectiveness [2, 4]. Therefore, if the existing cost-effective analysis already favours the current LTBI screening programme, then screening of the incident dialysis population would actually be cheaper and more cost-effective. As different renal units have varying screening protocols and TB incidences, a retrospective cost-effectiveness analysis to confirm these extrapolated conclusions could be performed as a research priority [42].

Information can also be inferred from cost-effectiveness analyses in Canada, which has a similar healthcare system to the UK. In British Columbia, Canada, it is routine practice to screen all pre-dialysis CKD patients for TB using either the TST or IGRA tests. Patients found to be positive for these tests with no evidence of active TB disease on assessment were treated for LTBI with a 9-month course of isoniazid. A cost-effectiveness analysis of this screening programme found that IGRA testing was more cost-effective than TST, and that screening for LTBI was only cost-effective (defined as cost per quality adjusted life year <$48 000) in patients with advanced CKD who were from high incidence countries and over the age of 60 years [43]. Breaking with convention, this study suggests that elderly CKD patients may receive more benefit from LTBI chemoprophylaxis than younger patients or those with less advanced kidney disease as they are likely to undergo reactivation and develop more severe disease if this occurs.

### Patient perspectives and acceptability of screening

The benefit of screening to the wider population is likely to be substantial, but understanding the benefit to the individual patient with CKD is less clear. Patient reported outcome measures are an increasingly important way of understanding the effects of diagnoses and treatments on a person's quality of life. A systematic review and meta-analysis of patient-reported outcome measures in LTBI showed that patients had similar measures of physical wellbeing to healthy controls [44]. However, they had worse measures of mental health, citing anxiety and stigma associated with the diagnosis of LTBI. It is therefore important that patients are only screened for LTBI if treatment can subsequently be offered and therefore the effect of LTBI treatment on quality of life needs to be ascertained. To date, there are no patient-reported outcome measures of LTBI treatment in CKD.

As stated previously, there is an increased risk of adverse drug effects with age and decreasing renal function [45]. A retrospective cohort study from British Columbia, suggests that 21% of CKD patients treated for LTBI experienced a grade 3–4 adverse event (most commonly gastrointestinal disturbance, general malaise and pruritus), and 0.5% of patients required hospitalization. There were no deaths associated with treatment [46]. Compared with a recent study in India analysing adverse drug reactions in patients with active TB, the risk of adverse events during treatment for LTBI is lower (risk of grade 3–4 adverse events in the Canadian LTBI group [46] 21% vs 33% in the Indian active TB group [45]). Given the significantly increased risk of LTBI progression to active TB in CKD, LTBI treatment to abrogate this risk is not only in the best interests of the patient but is also associated with fewer adverse events. This strategy is reinforced by the British Columbia experience whereby their LTBI screening programme in the dialysis population has reduced active TB incidence rates by 70% compared with dialysis patients not in the screening programme [47]. For those on dialysis diagnosed with active TB, the median time from dialysis initiation to active TB diagnosis was 300 days (interquartile range 81–420) [47], suggesting individual patients are likely to see benefit from reduced rates of active TB infection within their lifetime, and LTBI treatment is not just for the wider public health benefit. Studies from China further reinforce these findings: in a retrospective cohort study of 270 dialysis patients, IGRA testing was used to identify dialysis patients with LTBI [48]. After a median follow-up time of 39 months, 6.4% of patients with a positive IGRA test developed active TB compared with none of the IGRA negative patients. Furthermore, LTBI detection was significantly associated with major adverse cardiovascular events even when adjusting for sex, age, tobacco use, diabetes mellitus, pre-existing cardiovascular disease, anaemia and hypoalbuminaemia. This suggests there is benefit in treating dialysis patients with LTBI that goes beyond simply the prevention of active TB disease [48].

In summary, these data suggest that screening the entire pre-dialysis CKD population, including those >60 years old, is likely to benefit the individual, in addition to the strong public health argument. While adverse events are relatively common in this population, no deaths were recorded and they are arguably less than the risk of adverse effects associated with active TB treatment or major adverse cardiovascular events. To date, there are no studies investigating patient-reported outcomes of LTBI treatment in the CKD population and this should be an area prioritized for research to help further characterize the acceptability of treatment to patients.

### The use of artificial intelligence

The use of artificial intelligence (AI) and machine learning models to analyse large datasets from CKD and TB cohorts may also help to refine risk stratification, identifying patients who are most likely to benefit from LTBI screening and treatment [49]. These technologies could integrate clinical, demographic and laboratory data to predict TB reactivation risk, thereby optimizing screening programs and reducing unnecessary treatment in lower risk groups. For example, machine learning has been recently used to analyse a flow cytometry panel measuring cytokine release from peripheral blood mononuclear cells to identify cytokine signatures that accurately correlate with LTBI at high risk of reactivation, although this model is yet to be validated in the CKD population [50]. This has recently been extensively reviewed elsewhere [49], and a greater understanding of the immunological mechanisms underpinning LTBI reactivation, especially in the context of CKD, is required to develop appropriate algorithms.

## CURRENT UK PRACTICE

**Figure 1:**
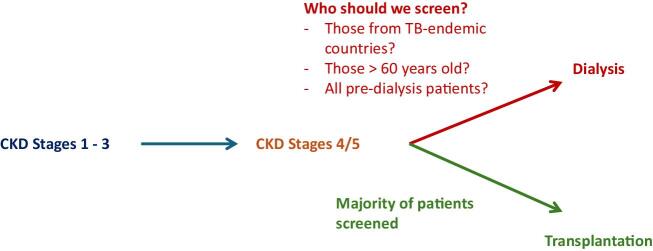
Evidence-based consensus is required on screening strategies for LTBI In the CKD population.

Current UK practice is informed by the National Institute for Health and Care Excellence (NICE) guidelines [51] and guidance from the British Thoracic Society (BTS) in 2010 [25], which has changed little despite a review by the authors in 2017 [52]. NICE recommend that patients who are immunocompromised should be actively screened for LTBI using either TST or IGRA. However, CKD is not listed as a potential high-risk condition, and there is national variation in how this guidance is interpreted. Guidance from the BTS is more specific and advises against universally screening all advanced CKD patients in favour of targeting high risk individuals and those being worked up for transplant. This recommendation is made on low quality grade D (expert opinion) evidence and attempts to balance the benefit of screening with the cost associated with testing all advanced CKD patients. This conclusion is in contrast to the 2018 WHO guidelines on LTBI which explicitly state that all dialysis patients should be screened for LTBI [32]. Nevertheless, there is a noteworthy lack of evidence informing practice in this area and therefore individual renal units across the UK have taken different approaches (Fig. 1).

We surveyed all renal units across England to explore variation in practice in LTBI screening. A 10-point survey (Supplementary data, Table S1) was sent out to the clinical service leads and/or consultant nephrologists responsible for infectious diseases at 56 renal units. Of those contacted, 36 (64.2%) responded giving us a strong representation of practice nationally (Supplementary data, Table S2). Survey uptake was poor in some regions with lower incidence rates of TB (Table 2).

**Table 2:** Renal unit survey response rates for 56 units contacted across all English regions.

					Screening tool used (%)		
					
Geographical region	Annual Number of Incident TB Cases in General Population (Average)	RRT prevalence	Renal unit response rate (% of region)	Formal LTBI policy in use (%)	IGRA	TST	CXR and/or Hx with Ex
Midlands	1876	11602	8 (72.7)	45.4	100	0	54.5
East of England	762	4961	3 (42.9)	33.3	66.7	33.3	100
London	3201	15389	6 (85.7)	33.3	83.3	0	83
North East England	178	2762	2 (66.7)	100.0	50	0	50
North West England	1043	6966	6 (85.7)	50	100	0	100
South East England	1015	7328	6 (100)	33.3	83.3	16.7	66.7
South West England	356	5015	2 (28.6)	50.0	100	0	100
Yorkshire and the Humber	661	6022	3 (42.5)	66.7	100	0	33.3
All regions	9092	60045	36 (64.2)	48.6	83.8	5.4	75.7

Outlines the incidence of new TB cases per region based on previously published averages of 2021–2023 data alongside prevalence of patients requiring RRT [20, 38]. Also outlines percentage of responding units with a formal LTBI screening policy in place and general screening tools used.

CXR, chest X-ray; Ex, examination; Hx, history.

**Figure 2:**
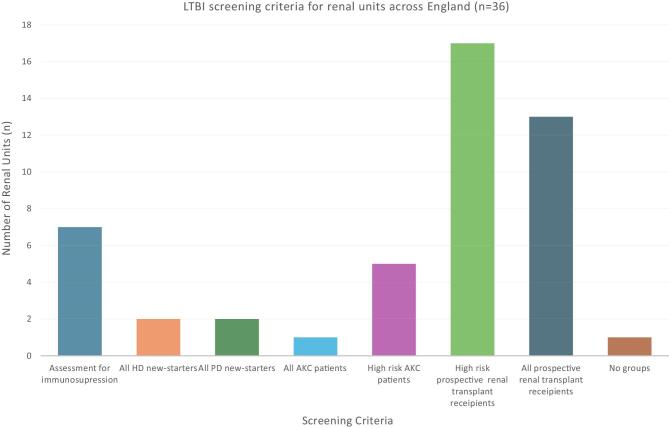
Outline of the selection criteria used for LTBI screening at renal units across England. High-risk indicates patients with medical history of TB or born in endemic regions. HD, haemodialysis; PD, peritoneal dialysis.

Less than 50% of renal units report having a formal LBTI screening protocol in place. This includes three regions with the highest incidence of TB: London, the Midlands and North West England. Aside from one unit based in London, all report some form of routine screening that target specific populations in their catchment area (Fig. 2). As evident in Fig. 2, there is no standardized approach to targeting patient groups. Not only is there variation between regions, but there are also intra-regional variations in screening targets. In London, there is a unit that screens all new-starters on renal replacement therapy (RRT) and plan to extend this practice to all Advanced Kidney Care Clinic (AKC) patients approaching the need for dialysis. Yet another unit less than 6 kilometres away does not routinely screen their patients for LTBI. There is even variation within trusts. Two trusts in the north west and midlands have one unit screening high risk AKC cases alongside prospective transplant cases whilst their counterparts in another unit routinely screen prospective transplant cases only (Supplementary data, Table S3).

### Ethical and cost considerations of variation in screening practices

The survey demonstrates a wide variety in screening practices nationally. Many centres use country of origin as a key inclusion factor in screening protocols. There is a risk that this strategy can contribute to stigma for those who have migrated to the UK. Since 80% of active TB cases in England in 2023 were identified in people born outside the UK [20], birth in a TB endemic country is therefore an established risk factor for TB and screening with informed consent in this population is important for the health of this demographic group. However, the TB notification rate for people born within the UK increased by 5% in 2022 [20]—the greatest increase in 10 years—and therefore while country of origin is an important risk factor, it should not preclude appropriate TB screening in the UK-born population where TB incidence is also rising.

Reducing stigma around LTBI testing for immigrants requires reframing the narrative to emphasize public health benefits rather than individual risk, using culturally sensitive communication, and integrating testing into routine healthcare to normalize it. Confidentiality and non-punitive policies should be reinforced to ease fears around immigration status. Engaging community leaders, peer ambassadors, and trusted institutions can help build trust and dispel misconceptions. Healthcare providers should receive training in non-stigmatizing discussions. Finally, policy changes that improve accessibility, remove reporting barriers and integrate TB services into primary care can further support a stigma-free approach.

The variation in screening practices needs further comparison and auditing to determine whether outcomes differ significantly between centres. For example, the estimated TB incidence amongst dialysis patients in Birmingham is 256 per 100 000 persons/year but is predicted to be 600 per 100 000 persons/year in Leicester. This difference in incidence may be due to the distinctive ethnic composition of these two diverse cities [53], or it could be because Leicester's screening programme is more comprehensive and therefore detects more cases. Leicester is the only Midlands unit which screens all their advanced CKD patients and new starters on dialysis for TB. A retrospective cohort study comparing these centres could therefore facilitate much needed cost-effectiveness analyses, assess the incidence of LTBI and active TB cases, and collect patient reported outcome measures which would bring valuable additional data to this area.

## GLOBAL PRACTICE

The WHO recommends that all pre-dialysis CKD patients, and those newly starting dialysis, should be screened for LTBI. The ECDC also advocates LTBI screening across the European Union for all patients commencing or approaching end-stage renal failure requiring dialysis after reviewing the available studies [34, 54–56]. This is based on evidence that, while the prevalence of LTBI is not different to the general population, the risk of future conversion to active TB is significantly increased with a pooled relative risk of conversion in the dialysis population of 703.2 (95% confidence interval 38.1–12 984.5) relative to the general population [34]. The ECDC acknowledges that if end-stage renal failure patients do develop active TB, they are likely to have more severe disease and poorer outcomes, thereby reaffirming its decision to endorse screening. The cost-effectiveness analyses reviewed by the ECDC also conclude that screening high-risk groups such as the pre-dialysis population using IGRA testing is cost-effective, although the available evidence did not specifically look at the dialysis/CKD population [55]. As already outlined in this review, Canada also has a comprehensive screening strategy for its dialysis population [33] which has been shown to be cost-effective and has led to a reduction in active TB cases [47].

In lower and middle income countries, the limited availability of IGRA testing means there is significant variation in practice and explains WHO's recommendation to use TST or IGRA in LTBI screening [32]. In India the TST is widely used due to its availability [57], but this is fraught with issues in the CKD population that have been detailed in large cohort studies from the Indian subcontinent [27].

## NOVEL APPROACHES AND FUTURE DIRECTIONS

There are several research questions which need urgently addressing to improve the care provided to patients in this growing syndemic.

Firstly, the diagnostic tools available are unsatisfactory and the development of more sensitive and specific tests for LTBI are urgently needed. Urinary tests are being developed to detect lipoarabinomannan (LAM) produced by TB, but this is not suitable for use in advanced CKD and dialysis patients, where many patients are oligoanuric [58]. It is also not clear whether these tests are accurate in diagnosing LTBI or active TB. Blood tests attempting to identify changes in T-cell subsets in response to LTBI, as well as micro-RNA and mycobacterial DNA in the blood are being investigated [59]. Finally, there is interest in being able to detect virulence factors, such as ESAT-6, which may help to predict which patients with LTBI are likely to progress to active TB disease [59]. Stool tests and tongue swabs are also being investigated, although making a lateral flow test for TB is much more difficult than it was for detecting SARS-CoV-2 [58, 60]. Multiparameter flow cytometry shows promise for helping to differentiate between LTBI and active TB but these panels have not been validated in the context of CKD which has a known phenotype of immune dysfunction [50]. Researchers are also exploring the use of RNA sequencing and machine learning to identify genetic signatures of active TB and LTBI. However, patients with end stage renal disease have been excluded from the study's recruitment [61]. Indeed, none of the prospective novel tests has been trialled in the CKD population and therefore novel diagnostics remain elusive.

Secondly, better quality evidence is required to understand which subpopulations of the CKD cohort would truly benefit from LTBI screening. The current status quo is built upon poor quality evidence and expert opinion which seeks to balance cost with patient benefit. A comparative study of outcomes between UK renal centres could help to address this unmet need for further data. The discrepancy in TB incidence highlighted here suggests that the extent of disease burden may be significantly underestimated.

## CONCLUSION

Evidence from different global settings suggests that LTBI screening in specific subgroups of the CKD cohort would confer benefit. The WHO and many other high-income countries with similar healthcare systems to the UK advocate screening for LTBI in the pre-dialysis and RRT new starter populations. Based on the current evidence and information from the annual UK Tuberculosis Report, we also advocate this approach, accepting that the current diagnostics are suboptimal and further research is required in this area. Comparing outcomes across UK renal centres with divergent screening policies would provide important evidence to drive recommendations, and a data linkage study combining data from the UK Tuberculosis Report and the UK Renal Registry would further strengthen the evidence base for recommendations.

Emerging diagnostics and AI-driven approaches hold promise for improving screening and treatment strategies, but more high-quality evidence is needed to guide clinical practice. The magnitude of this syndemic is only predicted to grow in the coming years and answering these questions should therefore be a research priority.

In conclusion, patients with CKD, particularly those with ESRD requiring dialysis, face a substantially heightened risk of TB reactivation. However, the identification and management of LTBI in this population remains complex and challenging. Existing diagnostic tools lack accuracy in immunocompromised patients, leading to potential underdiagnosis or overtreatment. Furthermore, the decision to initiate LTBI therapy must carefully weigh the risks—such as drug toxicity and interactions—against the benefits, especially in older patients with multiple comorbidities. Addressing these challenges requires more precise diagnostic strategies, individualized risk assessment and a multidisciplinary approach to optimize patient outcomes.

## Supplementary Material

sfaf197_Supplemental_Files

## Data Availability

The data underlying this article will be shared on reasonable request to the corresponding author.
