# Supplementary material for: Screening for latent tuberculosis infection in patients with chronic kidney disease: a review of evidence and current practice in the UK
Source: Clin Kidney J. 2025 Jun 19;18(7):sfaf197. doi: 10.1093/ckj/sfaf197 (PMC12378438; doi:10.1093/ckj/sfaf197)
Supplement: sfaf197_Supplemental_Files [file sfaf197_Supplemental_Files.zip › 121 Supplementary tables.docx]

# Supplementary tables:

| Question | Answer |
| --- | --- |
| Please enter your name | - Free text |
| Please enter your role in your unit with regards to TB | - Free text |
| Renal Unit/Hospital Name | - Free text |
| Town/City | - Free text |
| Does your unit have a formal policy on when to screen for latent TB infection? | - Yes or No |
| If you have answered no to question 3, please outline how practice varies in your unit: | - Free text |
| In your unit, when do you routinely screen for latent TB infection? (please tick all that apply) | Multiple choice selection:   - All patients in the general nephrology clinic - All patients in the Advanced Kidney Care Clinic (AKC), regardless of risk profile - AKC patients deemed high risk based on country of birth and medical history - All patients with CKD G4/5 - All prospective kidney transplant recipients, regardless of country of birth or risk profile - Prospective kidney transplant recipients identified as high risk based on country of birth and medical history - All new starters on haemodialysis (HD), regardless of risk profile - New starters on HD identified as high risk based on country of birth and medical history - All new starters on peritoneal dialysis (PD), regardless of risk profile - New starters on PD identified as high risk based on country of birth and medical history - Other (please specify further) |
| What tests do you routinely use to screen for latent TB infection? (please tick all that apply) | Multiple choice selection:   - History and examination - Chest X-ray - Tuberculin Skin Test (i.e.- Mantoux Test) - Interferon Gamma Release Assays (i.e. - Quantiferon Gold, T-Spot) - Other (please specify) |
| If a patient tests positive for potential latent TB, who does the patient get referred to for further investigation and management? | Multiple choice selection   - In-house TB renal clinic - Respiratory Medicine - Infectious Diseases - Other (please specify) |

*Supplementary Table 1: 9-point survey sent to all Renal units in England using SurveyMonkey®*

| **Region** | **Renal Unit/Hospital (n=36)** |
| --- | --- |
| London | King's College Hospital |
|  | West London Renal and Transplant Centre |
|  | Royal Free Hospital |
|  | St George's Hospital |
|  | Barts Health NHS trust |
|  | St Helier renal Unit |
| North West | Salford Royal |
|  | NHS University Hospitals of Liverpool Group (2 Responses) |
|  | Manchester Royal Infirmary |
|  | Cumberland Infirmary |
|  | Wirral University Teaching Hospital |
|  | Royal Preston Hospital |
| Yorkshire and the Humber | Sheffield Teaching Hospitals |
|  | Bradford Teaching Hospitals |
|  | Doncaster Royal Infirmary |
| North East | Freeman Hospital, Newcastle |
|  | Sunderland Hospitals |
| Midlands | Queen Elizabeth Hospital, Birmingham |
|  | Birmingham Heartlands Hospital |
|  | New Cross Hospital, Wolverhampton |
|  | Shrewsbury Hospital |
|  | University Hospitals Conventry & Warwickshire |
|  | University Hospitals of Leicester NHS Trust |
|  | Nottingham City Hospital |
|  | University Hospitals of Derby and Burton |
| South East | East Kent Hospital University NHS Foundation Trust |
|  | Royal Berkshire Hospital |
|  | University Hospitals Sussex |
|  | Queen Alexandra Hospital |
|  | Lister Hospital, Stevenage |
|  | Churchill Hospital (Oxford University Hospitals) |
| East of England | Norfolk and Norwich University Hospital |
|  | Mid and South Essex Hospital |
|  | Cambridge University Hospitals |
| South West | University Hospitals Plymouth |
|  | Gloucestershire Hospitals |

*Supplementary table 2: Outlines renal units that responded from each region of England.*

*Supplementary table 3: See raw data supplementary excel file for survey response data. Unit and responding clinician have been anonymised.*
